# Supplementary material for: The human-specific paralogs SRGAP2B and SRGAP2C differentially modulate SRGAP2A-dependent synaptic development
Source: Sci Rep. 2019 Dec 10;9:18692. doi: 10.1038/s41598-019-54887-4 (PMC6904453; doi:10.1038/s41598-019-54887-4)
Supplement: Supplementary file 1 — Supplementary Material [file 41598_2019_54887_MOESM1_ESM.pdf]

Supplementary material

The human-specific paralogs SRGAP2B and SRGAP2C differentially modulate SRGAP2A-dependent synaptic development

Ewoud R.E. Schmidt, Justine V. Kupferman, Michelle Stackmann, and Franck Polleux

6 Supplementary Figures and 1 Supplementary Table

Figure S1

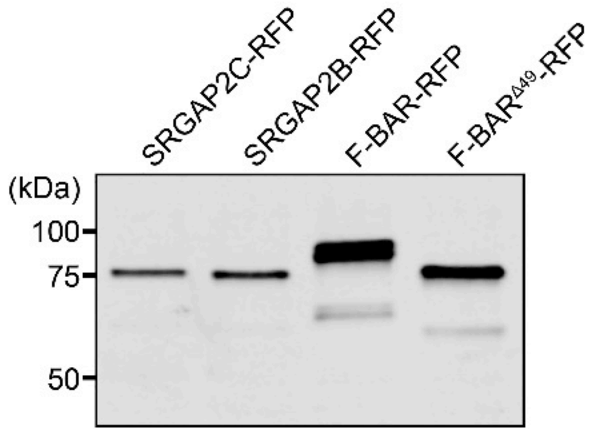

Figure S1. Testing expression of fusion proteins in Neuro-2a cells.

Western blot showing expression of indicated mRFP-fusion proteins in Neuro-2a cell lines. SRGAP2C-RFP, SRGAP2B-RFP, and F-BAR$^{\Delta 49}$-RFP run slightly lower than F-BAR-RFP due to their lack of the last C-terminal 49 amino acids.

Figure S2

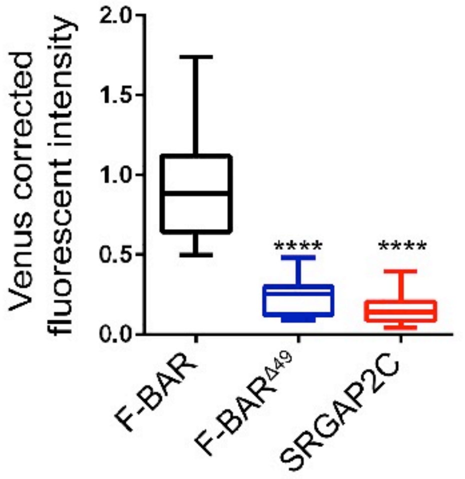

Figure S2. Expression levels for F-BAR proteins in cortical pyramidal neurons.

Box plots showing quantification of mRFP-tagged F-BAR, F-BAR$^{\Delta49}$, and SRGAP2C fluorescent intensity in the soma of cultured cortical pyramidal neurons. $n_{F\text{-}BAR} = 21$, $n_{F\text{-}BAR^{\Delta49}} = 33$, $n_{SRGAP2C} = 31$; ****$p < 0.0001$.

Figure S3

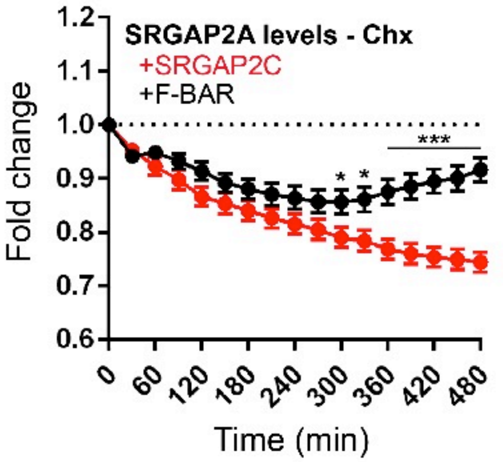

Figure S3. SRGAP2A turnover when co-expressed with SRGAP2C.

Quantification of SRGAP2A fluorescent intensity upon treatment with cycloheximide (Chx). When co-expressed with SRGAP2C, the reduction of SRGAP2A levels is significantly increased compared to co-expression with F-BAR. $n_{F\text{-}BAR} = 24$, $n_{SRGAP2C} = 29$; *$p < 0.05$, ***$p < 0.001$; mean ± SEM.

Figure S4

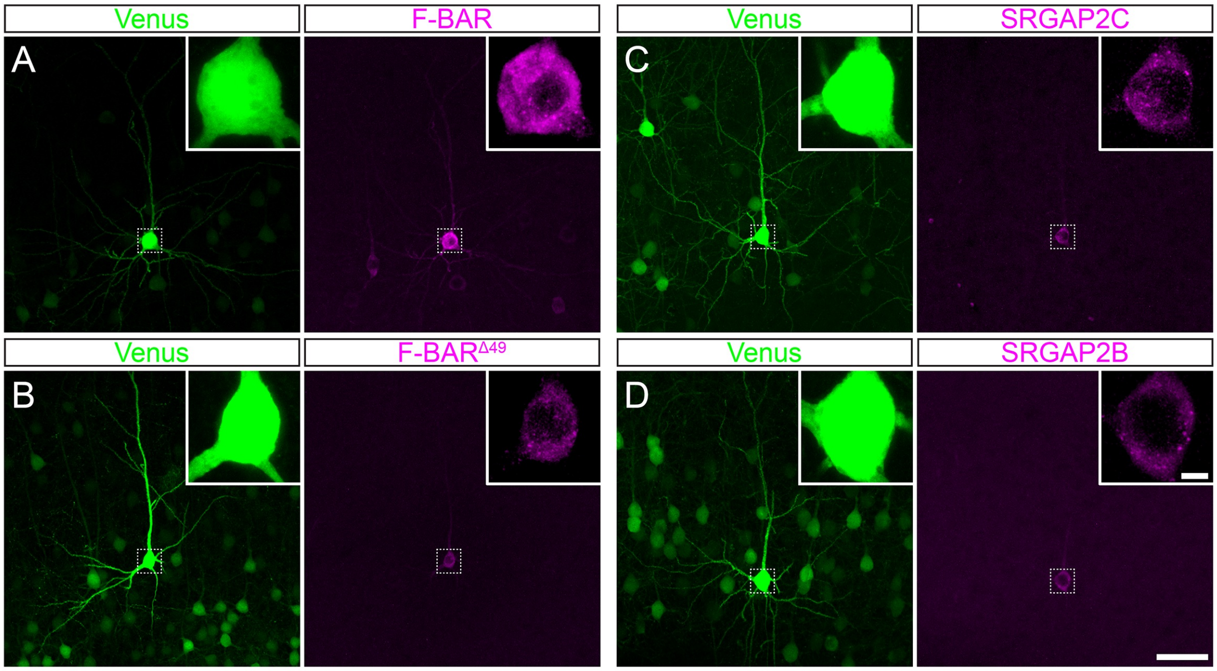

Figure S4. *In vivo* expression of F-BAR proteins in layer 2/3 cortical pyramidal neurons.

Representative images showing *in utero* electroporated layer 2/3 cortical pyramidal neurons expressing Venus with either mRFP-tagged F-BAR, F-BAR$^{\Delta 49}$, SRGAP2B, or SRGAP2C. Insets show high magnification images of soma (indicated with dashed rectangle). Scale bars: 50 μm, and 5 μm for inset.

Figure S5

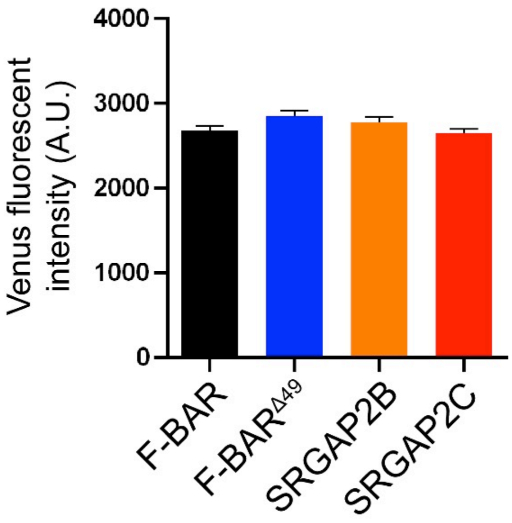

Figure S5. Expression levels in electroporated cortical neurons

Quantification of Venus optical density in dendritic spines of electroporated cortical pyramidal neurons for all four conditions (fusion proteins) used in the paper. No statistical difference was observed between any of the constructs. Number of spines: $n_{F\text{-}BAR}$ = 1305, $n_{F\text{-}BAR^{\Delta 49}}$ = 1359, $n_{SRGAP2B}$ = 1148, $n_{SRGAP2C}$ = 1308; one-way ANOVA with Holm-Sidak's *post hoc* multiple comparison test.

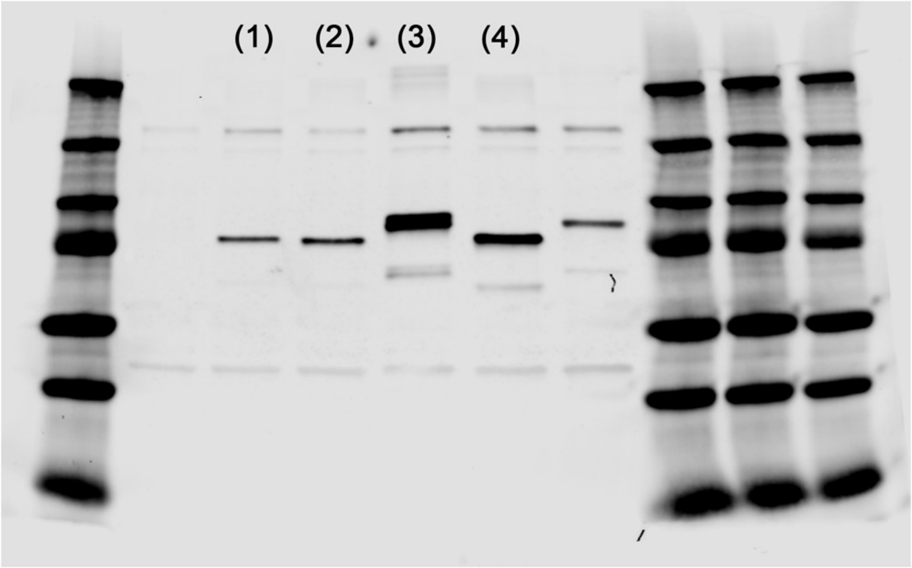

1) SRGAP2C-RFP
2) SRGAP2B-RFP
3) F-BAR-RFP
4) F-BAR$^{\Delta49}$-RFP

Figure S6. Full western blot shown in Figure S1.

Table S1

	Age	Mice	Neurons	Dendrites	Spines	Density	Size
Control	P21	7	18	25	1693	0.95	0.44
F-BAR	P21	5	19	25	2054	1.19	0.36
F-BAR$^{\Delta49}$	P21	5	23	27	3267	1.45	0.37
SRGAP2B	P21	6	19	25	2501	1.43	0.35
SRGAP2C	P21	5	19	25	2448	1.64	0.31
Control	>P65	5	23	25	1872	1.25	0.43
F-BAR	>P65	4	24	24	2049	1.21	0.41
F-BAR$^{\Delta49}$	>P65	4	15	22	1843	1.25	0.38
SRGAP2B	>P65	4	16	20	2295	1.34	0.39
SRGAP2C	>P65	6	18	25	2630	1.59	0.40
	**Totals**	**51**	**194**	**243**	**22,652**		

Table S1. Number of mice, neurons, branches, and dendritic spines analyzed for each condition for the experiments in Figures 3 and 4. Values for mean spine density (spines/um) and spine size (um^2) are listed for each condition.
